# Re: Zhang Y, Long G, Shang H, Ding B, Sun G, Ouyang W, et al. Comparison of the oncological, perioperative and functional outcomes of partial nephrectomy versus radicalnephrectomy for clinical T1b renal cellcarcinoma: a systematic review and meta-analysis of retrospective studies. Asian J Urol 2021;8: 117–25. Can we improve renal function by partial nephrectomy?

**DOI:** 10.1016/j.ajur.2021.04.004

**Published:** 2021-04-24

**Authors:** Samir Bouras, Samir Yebdri, Kamal Adjali

**Affiliations:** Department of Urology, Faculty of Medicine, Ferhat Abbas University Setif 1, Setif, Algeria; Department of Urology, Faculty of Medicine, Mouloud Mammeri University, Tizi OuZou, Algeria; Department of Urology, Faculty of Medicine, Benyoyucef Benkhedda University Algiers 1, Algiers, Algeria

To the editor:

I had the pleasure of reading a very interesting, original article published recently by Zhang et al. [1] in your prestigious journal, entitled “Comparison of the oncological, perioperative and functional outcomes of partial nephrectomy versus radical nephrectomy for clinical T1b renal cell carcinoma: A systematic review and meta-analysis of retrospective studies”. The interest in partial nephrectomy (PN) remains relevant after all these years of standardizing PN for cT1 renal masses [2,3]. As all similar publications, this technique cannot be talked about without praising it for the preservation of long-term renal function (RF) compared to radical nephrectomy, especially for patients with a chronic kidney disease (CKD) [[1], [2], [3]]. This article contains no less than two references whose titles refer directly to the RF. The interest in this aspect comes from our thesis work on PN, where we noticed an improvement in postoperative RF and its maintenance over time, in some patients, even with CKD, whether in our study or elsewhere.

It is well known that early postoperative RF depends essentially on modifiable factors, in particular the duration of ischemia [4], which can magnify the trauma effect, including the disruption of osmotic gradient by localized edema, the cytokine inhibitory effect, and microvascular blood flow alteration [5].

While this potentially deleterious effect is reversible within a few weeks to a few months, several risk factors have been described: Advanced age, significant comorbidities, preoperative CKD, and nephronic loss which is permanent, as a result of excision of the tumor in healthy margins, and collateral damages caused by reconstruction (hemostasis and nephrorraphy) inducing compression and necrosis, in particular with pyramidal atrophy by ligation of the calyces.

Thus, long-term RF depends mainly on the amount of preserved or remaining parenchyma [4,5]. With two kidneys, the functional loss of the operated kidney is estimated at 20%. Furthermore, given that the compensatory hypertrophy of the contralateral kidney after PN in adults is insignificant (marginal), the overall loss of RF is estimated to be 10% [4]. From now on, the loss of RF is predictable and reducing this loss as much as possible, through an appropriate medical and surgical approach, should be a priority. What is unpredictable and of particular interest to us, is that among all these patients, some will see their RFs improve postoperatively. Uro-pediatricians have studied this subject in children who have had PN for non-syndromic Wilms tumors, assuming the existence of an unclear physiological mechanism by which the tumor can affect the RF preoperatively. However, it is necessary to preserve enough nephrons to hope for a compensatory hyperfunction.

Indeed, nephrectomized patients with CKD II did not improve their RFs unlike patients with CKD III who had PN [6]. While this explanation partially answers this question in children, compensation in adults is marginal [4]; the more so this improvement is often even short-term. This is confirmed by the results of Imran Khan et al. [7] in a very interesting study, comparing the results of PN on RF with or without pedicle clamping. They noticed an increase in the postoperative RF (group without clamping) at 3 months compared to the preoperative RF (93.14±40.70 *vs.* 88.00±36.11 mL/min) before decreasing at 1 year (91.93±39.60 mL/min). This study clearly showed that the effect of compensatory hypertrophy even after a few months could not explain the spectacular improvement in RF in the short-term. This is best seen on single kidneys. Indeed, Fergany et al. [8] had improvement of RF in 38% of their patients. Among the hypotheses which seem to join those of the uro-pediatricians, Sankina et al. [9] have suggested a possible local toxic effect by the tumor on the ipsilateral kidney, and that tumor excision cancels out this effect.

In addition, we know that most of the operated patients have a malignant pathology, which is for some a factor of poor functional results. The hypothesis put forward is a neovascularization which can be complicated by arteriovenous fistulas and thus lead to renal dysfunction [10]. We believe that tumor excision may partly explain this improvement.

Finally, although an improvement in RF has been observed in several studies, no such study has been published to our knowledge. We believe that the limited number of these patients hinders any exploration. We consider that an adequate assessment of these patients by establishing a careful epidemiological profile, and by analyzing risk factors, perioperative conditions, certain biological factors, and in particular tumor molecular biomarkers, is essential. The objective is not only to determine the risk factors for degradation of the RF, but above all to determine the factors for improvement of the RF, and thus to establish the groups of patients with good or better functional prognosis.

With incidence rates rising steadily around the world, we believe this topic deserves a research project. Unfortunately, at our level, we have neither the desired number of patients, nor the technology allowing in particular the biomolecular exploration which seems useful to us.

## Author contributions

*Study concept and design:* Samir Bouras, Kamal Adjali.

*Data acquisition:* Samir Bouras, Samir Yebdri.

*Data analysis:* Samir Bouras, Kamal Adjali.

*Drafting of manuscript:* Samir Bouras, Samir Yebdri.

*Critical revision of the manuscript:* Samir Bouras, Samir Yebdri.

## Conflicts of interest

The authors declare no conflict of interest.
